# m7G-related gene NUDT4 as a novel biomarker promoting cancer cell proliferation in lung adenocarcinoma

**DOI:** 10.3389/fonc.2022.1055605

**Published:** 2023-01-24

**Authors:** Yafei Liu, Bin Jiang, Chunjie Lin, Wanyinhui Zhu, Dingrui Chen, Yinuo Sheng, Zhiling Lou, Zhiheng Ji, Chuanqiang Wu, Ming Wu

**Affiliations:** ^1^ Department of Thoracic Surgery, The Second Affiliated Hospital Zhejiang University School of Medicine, Hang Zhou, China; ^2^ Life Sciences Institute, Zhejiang University, Hang Zhou, China

**Keywords:** N7-methylguanosine modification, lung adenocarcinoma, prognosis, NUDT4, immune infiltration, immunotherapy

## Abstract

**Background:**

Lung cancer is the leading cause of mortality in cancer patients. N7-methylguanosine (m7G) modification as a translational regulation pattern has been reported to participate in multiple types of cancer progression, but little is known in lung cancer. This study attempts to explore the role of m7G-related proteins in genetic and epigenetic variations in lung adenocarcinoma, and its relationship with clinical prognosis, immune infiltration, and immunotherapy.

**Methods:**

Sequencing data were obtained from the Genomic Data Commons (GDC) Data Portal and Gene Expression Omnibus (GEO) databases. Consensus clustering was utilized to distinguish m7G clusters, and responses to immunotherapy were also evaluated. Moreover, univariate and multivariate Cox and Least absolute shrinkage and selection operator LASSO Cox regression analyses were used to screen independent prognostic factors and generated risk scores for constructing a survival prediction model. Multiple cell types such as epithelial cells and immune cells were identified to verify the bulk RNA results. Short hairpin RNA (shRNA) Tet-on plasmids, Clustered Regularly Interspaced Short Palindromic Repeats CRISPR/Cas9 for knockout plasmids, and nucleoside diphosphate linked to moiety X-type motif 4 (NUDT4) overexpression plasmids were constructed to inhibit or promote tumor cell NUDT4 expression, then RT-qPCR, Cell Counting Kit-8 CCK8 proliferation assay, and Transwell assay were used to observe tumor cell biological functions.

**Results:**

Fifteen m7G-related genes were highly expressed in tumor samples, and 12 genes were associated with poor prognosis. m7G cluster-B had lower immune infiltration level, worse survival, and samples that predicted poor responses to immunotherapy. The multivariate Cox model showed that NUDT4 and WDR4 (WD repeat domain 4) were independent risk factors. Single-cell m7G gene set variation analysis (GSVA) scores also had a negative correlation tendency with immune infiltration level and T-cell Programmed Death-1 PD-1 expression, but the statistics were not significant. Knocking down and knocking out the NUDT4 expression significantly inhibited cell proliferation capability in A549 and H1299 cells. In contrast, overexpressing NUDT4 promoted tumor cell proliferation. However, there was no difference in migration capability in the knockdown, knockout, or overexpression groups.

**Conclusions:**

Our study revealed that m7G modification-related proteins are closely related to the tumor microenvironment, immune cell infiltration, responses to immunotherapy, and patients’ prognosis in lung adenocarcinoma and could be useful biomarkers for the identification of patients who could benefit from immunotherapy. The m7G modification protein NUDT4 may be a novel biomarker in promoting the progression of lung cancer.

## Background

In recent epidemiology statistics, lung cancer incidence showed a protracted downward trend, declining from 2009 to 2018 by almost 3% annually in men and 1% in women (1). The 5-year survival of lung cancer patients in the United States has improved from 17.2% 10 years ago to 21.7% in 2019 (2). It looks like there is some progress in lung cancer treatment, but an estimation about cancer deaths presented that lung cancer would cause a total of 66,820 men and 61,360 women to die in the year 2022, approximately 350 deaths per day (1). This overwhelming number reminds clinicians that there will always not (programmed death ligand 1) enough to study lung cancer. As radiographic technology has evolved and civilians’ health consciousness increased, more and earlier stages of lung cancer are being screened out (3), and diagnostic accuracy and recovery rate have improved in the past years, especially for patients diagnosed with stage IA, suitable for lung segmentectomy and lobectomy (4), which, undoubtedly, even increased the rate of lung cancer cure. Moreover, with applications of molecularly targeted drugs such as tyrosine kinase inhibitors and immune checkpoint PD-1/PD-L1 (programmed death ligand 1) inhibitors (5), patients in advanced stages had also improved survival outcomes, but as drug resistance inevitably occurs (6), patients’ conditions would suddenly deteriorate. Therefore, exploration of the mechanisms of tumor cell progression to find new drug targets is needed and worth to continue trying. On the other hand, constructing an efficient follow-up protocol for different recurrence risk levels is also needed for patients (7).

Small cell lung cancer and non-small cell lung cancer are the two main pathological classifications of lung cancer, and the latter accounts for approximately 85% of all lung cancer cases (8). Lung adenocarcinoma (LUAD) is one of the subtypes of non-small cell lung cancer, and which accounts for approximately 40% of all cases of lung cancer (9), diagnosed commonly in women, never smokers, and in East Asia (10). LUAD was demonstrated to transform from type II alveolar cell cancerization, which was corroborated by our previous study using single-cell sequencing analysis (11). The high probability of resistance to treatment and recurrence remains a major challenge for patients and clinicians. In this study, we would focus on N7-methylguanosine (m7G) modification-related characteristics in LUAD patients.

Since 1957, RNA modification has been discovered, and emerging multiple modification patterns have been identified, such as 1-methyladenosine (m1A), 5-methylcytidine (m5C), N6-methyladenosine (m6A), m7G, and 2-O-methylation, which affect RNA splicing, nucleation, and stability and have been involved in many eukaryote and prokaryote cell biological processes (12). In human diseases, RNA modifications were reported to have been involved in tumorigenesis, immunogenicity, and tumor immunity (13). As for lung cancer, RNA modifications have been widely reported to play an important role in tumor occurrence and development (14). Moreover, in the past decades, hundreds of enzymes that function as modifications have been found, which were capable of altering nucleosides in transfer RNAs (tRNAs), ribosomal RNAs (rRNAs), messenger RNAs (mRNAs), and microRNAs (15). These enzymes are of great potential to serve as diagnostic and prognostic biomarkers and therapeutic targets for the treatment of lung cancer.

m7G modification at nucleotide position 46 in the variable loop of tRNAs, as one of the most prevalent RNA modifications, has been reported to function as promoting the translation of cell cycle regulatory and oncogenic mRNAs and eventually driving cellular transformation and cancer progression (12). m7G-related proteins have been reported to be highly expressed in multiple cancer types and promote tumor development, such as lung cancer (16), hepatocellular carcinoma (17), prostate cancer (18), cholangiocarcinoma (19), and bladder cancer (20). However, the relationship between m7G modification proteins and the clinical characteristics of LUAD patients has not been properly evaluated. Therefore, in this study, we first used The Cancer Genome Atlas TCGA and Gene Expression Omnibus (GEO) sequencing data to analyze genetic variations in LUAD patients and then combined their clinical information to perform a Cox regression analysis to evaluate their impact on patients’ prognosis. In addition, we performed m7G consensus clustering and generated two clusters in which cluster B was highly expressed m7G-related genes, negatively correlated with immune infiltration, predicted poor responses to immunotherapy, and associated with poor survival outcomes. Thus, m7G-related proteins are of great potential as biomarkers and potential therapeutic targets in patients with LUAD.

The NUDT/(nudix hydrolase)NUDIX (nucleoside diphosphate linked to moiety X) type hydrolase superfamily included an evolutionary conserved large group of enzymes, which hydrolyzed a wide range of substrates, playing an important role in biological processes including cell proliferation, signal transduction, and homeostasis (21). The NUDIX-type motif 4 (NUDT4), being one of such enzymes, encoded protein diphosphoinositol polyphosphate phosphohydrolase 2 (DIPP2)whose function is to catalyze the reaction 7-methylguanosine 5′-triphospho-5′-polynucleotide + H2O = 7-methylguanosine 5′-phosphate + polynucleotide, thereby regulating the turnover of diphosphoinositol polyphosphates and as such, may also regulate vesicle trafficking and DNA repair (22, 23). It has been reported that NUDT4 is a prognostic biomarker in gastric cancer (24), but its role in lung cancer is also largely unknown.

## Methods

### Data acquisition and preprocessing

Copy number variation, simple nucleotide variation, transcriptome profiling, and clinical data were obtained from the National Cancer Institute Genomic Data Commons Data Portal, in which samples without survival time or survival status were excluded. Then, the transcriptome profiling gene IDs were converted from Ensembl ID to official gene symbols, and the normalization algorithm was transformed from fragments per kilobase of transcript per million fragments mapped FPKM to transcripts per million TPM for subsequent analysis. GEO dataset GSE68465 (25), GSE75037, GSE32863,GSE7670, GSE43458, including mRNA expression and clinical data, was also obtained. The RNA expression data Affymetrix probe IDs were also converted to official gene symbols. The transcriptome data from TCGA and GEO were merged with batch effect adjusted using empirical Bayes methods (26) for comprehensive analysis. Single-cell data were obtained from GEO dataset GSE131907 (27) and processed by R packages Seurat (28) and SingleR (29).

### Collection of m7G modification-related genes

According to the gene set enrichment analysis (GSEA) Molecular Signatures Database m7G-related gene sets (30) including gene sets GOMF m7G 5-PPPN diphosphatase activity, GOMF RNA 7-methylguanosine cap binding, GOMF RNA cap binding, and previously published articles (31–33), a total of 28 related genes were obtained for subsequent analysis, namely, AGO2 (argonaute 2), CYFIP1, DCP2, DCPS, EIF3D, EIF4A1, EIF4E, EIF4E1B, EIF4E2, EIF4E3, EIF4G3, GEMIN5, IFIT5, LARP1, LSM1, METTL1, NCBP1 (nuclear cap-binding protein 1), NCBP2, NCBP2L, NCBP3, NSUN2, NUDT10, NUDT11, NUDT16, NUDT3, NUDT4, SNUPN, and WDR4 (WD repeat domain 4).

### Mutation status and copy number variation analysis

The VarScan2 workflow-generated mutation data were used for further analysis. Sequencing data were loaded into RStudio software and analyzed by R package Maftools (34), which was capable of analyzing and visualizing the mutation percentage of the desired genes, variant classifications and types, variant allele frequencies, single-nucleotide variant (SNV) classes, and survival analysis between mutant and wild types of samples. Copy number variation data were visualized by the function barplot of the package Graphics in RStudio software (Version 1.4.1717).

### RNA expression comparison and survival analysis

Transcriptome data from TCGA tumor and normal samples were log2 ratio transformed for normalization before Wilcoxon test comparison using R package Limma (35), and the results were visualized by the function ggboxplot of the R package Ggpubr. In addition, data for survival analysis were merged from TCGA and GEO mRNA expression data, the method of which was mentioned above. After combination with clinical information, data were analyzed by the R package Survival and Survminer and visualized by Kaplan–Meier survival curves. Correlations between m7G genes were analyzed by the R package Psych using the Pearson method, and results were visualized by the R package Igraph.

### Evaluation of unsupervised consensus clustering, gene set variation analysis, and immunotherapy responses

To explore m7G molecular subclasses and analyze the characteristic differences in LUAD patients, mRNA expression data that only contained m7G-related genes were generated for consensus clustering. The R package ConsensusClusterPlus (36) was the tool used to subsample and determine the number of possible clusters in the dataset and provide heatmaps and distribution plots to help determine specified cluster counts. Moreover, the differentially expressed genes between clusters were analyzed, and gene set variation analysis (GSVA) (37) was performed to explore different pathways involved between clusters. Immunophenoscore (IPS) was defined as a digitized prediction of responsiveness to cytotoxic T-lymphocyte associated protein 4 CTLA-4 and PD-1 checkpoint inhibitors, which was obtained from The Cancer Immunome Atlas (TCIA). IPS comparison between m7G groups was analyzed and visualized by the Ggpubr package.

### Univariate Cox, LASSO Cox, and multivariate Cox regression analyses

Patient samples with m7G-related gene RNA expression and clinical characteristics including age, gender, and T and N stages were included in the analysis. Then, these factors were evaluated by univariate Cox analysis, and factors with a *P* < 0.05 were included in the LASSO Cox analysis. The R package Glmnet (38) was used for LASSO regression analysis, which helps to cross-validate each model to optimize the most fitted model. Eventually, the factors in the optimized model were included in the multivariate Cox regression analysis, and the regression model was visualized by nomogram using the package Rms, evaluated using receiver operating characteristic (ROC) curve by the TimeROC (39) package. The risk score was predicted by package Stats, and risk levels “High” and “Low” were divided by the median risk score.

### Single-cell RNA data processing and evaluation of immune infiltration levels

Patient sample types as primary tumor sequencing data from GSE131907 were downloaded, preprocessed, and integrated for further analysis according to the Seurat protocol. Data were imported and transformed into a Seurat project, in which cells with gene features >200 and <7,000 were obtained for further analysis, then the 11 samples were integrated into one Seurat project. Next, we used SingleR to identify cell types, which used Human Primary Cell Atlas (40), Blueprint (41), and ENCODE (42) as references. Cell types with small proportions were filtered out, and cell proportions including T cells, B cells, monocytes, and Natural killer NK cells were considered as tumor infiltration levels. Then, the GSVA scoring for m7G-related genes was performed on single-cell RNA data and visualized by boxplot.

### NUDT4 protein level expression from The Human Protein Atlas

To confirm whether the gene NUDT4 translates the associated protein in lung tissue cells, we used The Human Protein Atlas database (https://www.proteinatlas.org/) (43) to observe the protein expression both in LUAD and normal tissue chips. The proteins in human tissues based on immunohistochemistry using tissue microarrays, and antibodies including HPA017593, HPA057684.

### Cell culture

LUAD cell lines A549 and H1299, and 293T cells for generating lentivirus, were cultured in dulbecco's modified eagle medium DMEM with 10% fetal bovine serum (FBS) and 1% penicillin-streptomycin and placed in an incubator with 5% carbon dioxide at 37°C. Short hairpin RNA (shRNA) plasmids and envelope plasmids were cotransfected into 293T cells with polyethylenimine (PEI) transfection reagent, and after 48 h, the supernatant containing the lentivirus was collected to infect A549 and H1299 cells. Then, doxycycline was used to induce shRNA expression.

### Plasmid construction

The sequence for knocking down the NUDT4 expression was predicted from the Sigma website (https://www.sigmaaldrich.cn/CN/zh/product/sigma/shrna), including shNUDT4-1: CTCCAGTGTCATAAACCTGTA, shNUDT4-2: TTTGAGAACCAAGACCGAAAG, and shNUDT4-3: TCCCTTCCCTTCCGGATAATA, which were all cloned into tet-pLKO-puro. Tet-pLKO-puro empty plasmid was considered a negative control. On the other hand, three single-guide RNAs (sgRNAs) were designed to knock out NUDT4, including sgRNA1: TATCTGGAAAAGCTAAAGCT, sgRNA2: CACTGAAATATTAGAAGATT, and sgRNA3: CAGACTTCTGGGCATATTTG, which were all cloned into plasmid pX459. The primers used to clone the NUDT4 DNA are as follows: NUDT4sg12 (forward 5′-TTGCTTATATTCCTGTGTCCAGTTTCCATC-3′, reverse 5′-ATATAGGGGGTTGTGGGGTGTGGCACAGTT-3′); NUDT4sg3 (forward 5′-TCCAATAATTTGAGAGTTTTACCACCAGTAG-3′, reverse 5′-CATTTCAGTATCAGCTCCACTGTATTTTCAAAC-3′). For overexpression, the NUDT4 mRNA (GenBank: NM_019094.4) was expressed transiently in lung cancer cell lines using the pBOBI plasmid vector.

### Quantitative real-time PCR

Total cell RNA was extracted by TRIzol (Takara, Japan) reagent following the manufacturer’s instructions, 1 mg of which was used to synthesize the cDNA using the PrimeScript RT Master Mix (Takara, Japan). qRT-PCR assays were then performed utilizing SYBR Premix Ex Taq II (Takara, Japan). Glyceraldehyde-3-phosphate dehydrogenase GAPDH was chosen as an endogenous control to normalize the NUDT4 expression levels in different groups. The relative mRNA expression levels of NUDT4 were calculated using the 2^-ΔΔCt^ method. The primers used are as follows: GAPDH (forward 5′-GTCTCCTCTGACTTCAACAGCG-3′, reverse 5′-ACCACCCTGTTGCTGTAGCCAA-3′); NUDT4 (forward 5′-TACCCAGACCAGTGGATTGTCC-3′, reverse 5′-TGTTCTGTGCTTTCGGTCTTGGT-3′).

### Cell Counting Kit-8 assay

shRNA cell lines were seeded in 96-well plates at a density of 1,500 cells per well for A549 and 3,000 cells per well for H1299 and cultured for 3 days; knockout cell lines and overexpression cell lines were seeded at a density of 3,000 cells per well. Sample size in each experiment was designed as three duplicates. CCK8 reagent was used to evaluate cell proliferation in the indicated time points. Briefly, 10 µl of CCK8 reagent was added into each well in 96-well plates and then incubated for 4 h in an incubator at 37°C. Then, the plates were read at an optical density of 450 (OD450) nm by a microplate reader.

### Cell migration assay

Cell migration assays were performed using Transwell chambers (8-mm pore size) (NEST, USA). In this study, 4 × 10^4^ cells were suspended in serum-free medium and added to the upper face of the cell culture chambers. Sample size in each experiment was designed as three duplicates. The chambers were placed into a 24-well plate containing DMEM with 15% FBS. After incubation for 16 h, the chambers were fixed for 30 min using 4% polyoxymethylene (PFA), stained by crystal violet for 30 min, washed three times using Phosphate buffered solution PBS, and finally photographed with a microscope when the chamber became dry.

### Statistical analysis

All statistics were analyzed using R packages in RStudio software (R Version 4.1.2, RStudio Version 1.4.1717). The Wilcoxon test was used in mRNA expression comparison. The Pearson and Spearman method was used in correlation analysis. The Kaplan–Meier estimates were used in survival analysis. Univariate Cox, LASSO Cox, and multivariate Cox regression analyses were used to screen out independent risk factors. *P* < 0.05 was considered significant.

## Results

### Genetic alterations detected in m7G modification-related genes

**Figure 1:**
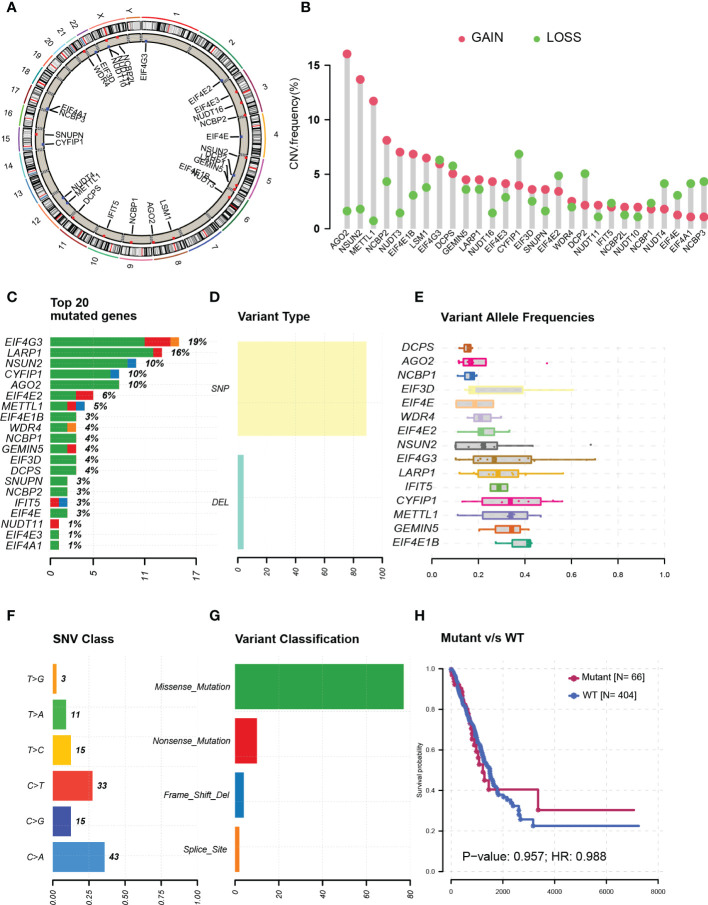
Genetic alterations in N7-methylguanosine (m7G) modification-related genes. **(A)** m7G modification-related genes’ chromosome locations. **(B)** m7G-related gene copy number amplification. **(C)** Top-rating mutated m7G-related genes. **(D)** Single-nucleotide polymorphisms in all variant types. **(E)** Variant allele frequencies of m7G-related genes. **(F)** Single-nucleotide variants of m7G-related genes. **(G)** Variant classification of m7G-related genes. **(H)** Survival analysis between mutant and wild-type patients. CNV: Copy number variation; SNP: Single-nucleotide polymorphism; DEL: Gene deletion; WT: Wild type; SNV: Single nucleotide variant; HR: Hazard ratio.

Genetic alteration data were only available in TCGA samples, which included 477 tumor tissue samples and 54 normal tissue samples. The chromosome locations of m7G modification-related genes were visualized on a chromosome ideogram (**Figure 1A**). Copy number amplification was observed mainly on genes AGO2, NSUN2, and METTL1 whose frequencies were all more than 10%. Most of the gene deletion frequencies were less than 5%, except the ones for genes CYFIP1, EIF4G3, and DCPS, which were a little higher than 5% (**Figure 1B**). On the other hand, genes with highest mutation rate were listed in **Figure 1C**, including EIF4G3 (19%), LARP1 (16%), and NSUN2 (10%). Single-nucleotide polymorphisms were the main variant types (**Figure 1D**). Moreover, almost all of the variant allele frequencies are less than 40% (**Figure 1E**). Most of the SNVs were from cytosine deoxynucleotide mutated to guanine or thymine deoxynucleotide (**Figure 1F**) and mainly missense mutations (**Figure 1G**). In addition, there was no difference in survival analysis between mutant and wild-type patients (*P* = 0.96) (**Figure 1H**).

### Most m7G-related genes were associated with poor prognosis

**Figure 2:**
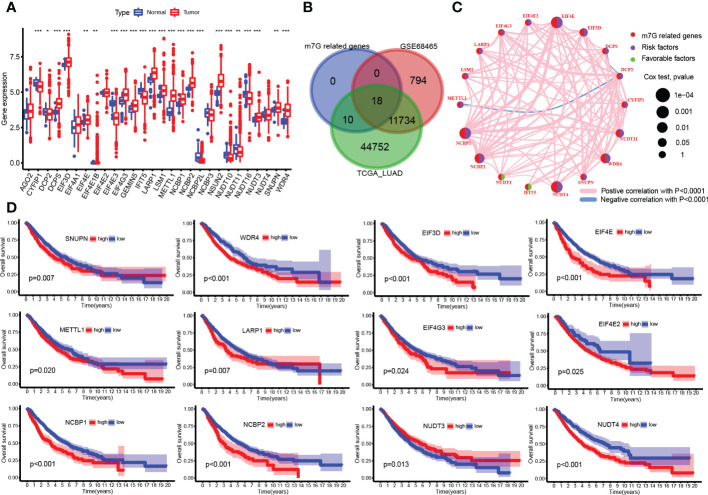
N7-methylguanosine (m7G)-related gene expression and survival analysis. **(A)** Boxplot of m7G-related gene mRNA expression between tumor and normal samples. **(B)** Venn plots for common existing genes in the three groups. **(C)** Correlation network between m7G-related genes. **(D)** Survival plot of m7G-related genes. (*P <0.05, **P < 0.01, ***P < 0.001, ns: Not statistically significant).

More than half of the m7G-related genes were significantly upregulated in tumor samples, such as METTL1, WDR4, EIF4E, LARP1, and LSM1 (**Figure 2A**). A total of 919 samples were left for analysis after samples without survival data were excluded, and after the integration of TCGA and GEO expression data, there were 18 genes left for survival analysis (**Figure 2B**). The correlation between each gene was also analyzed, which resulted in most of the genes being positively correlated with each other, but Decapping mRNA 2 DCP2 was negatively related to genes METTL1 and DCPS (**Figure 2C**). Fourteen genes were associated with patients’ poor prognosis, namely, WDR4, CYFIP1, DCP2, DIF3D, EIF4E, METTL1, LARP1, EIF4G3, EIF4E2, NCBP1, NCBP2, NUDT4, NUDT11, and SNUPN, but a high expression of NUDT3 was related to good survival in patients (**Figure 2D**).

### Two subclasses were clustered out by unsupervised consensus analysis

**Figure 3:**
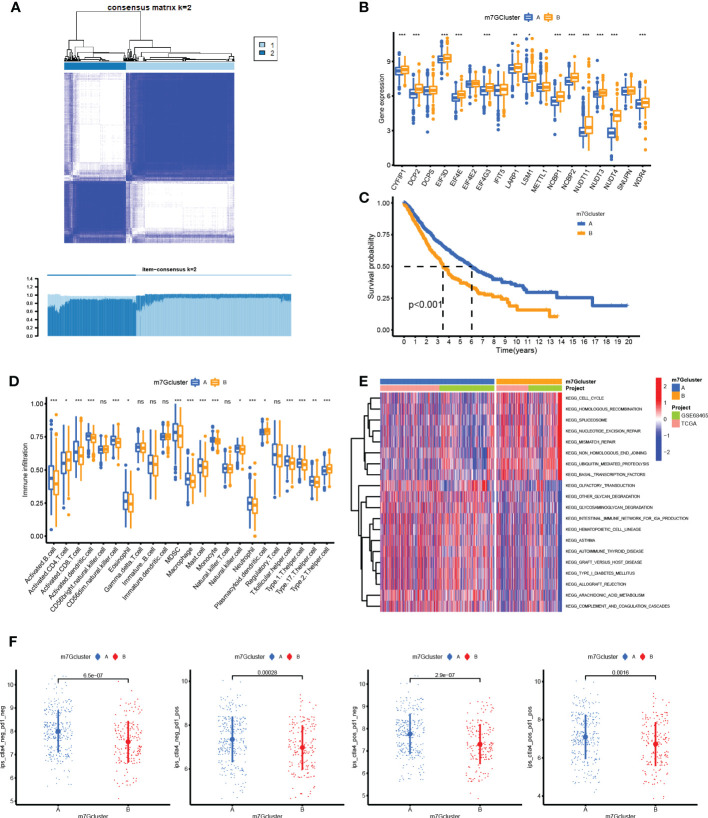
Unsupervised consensus clustering analysis. **(A)** Heatmap showed that the samples were distinctly divided into two clusters. **(B)** Boxplot of N7-methylguanosine (m7G)-related gene mRNA expression between the two clusters. **(C)** Survival plot for the two clusters. **(D)** Infiltration level of immune cells in different clusters. **(E)** Heatmap for KEGG pathway enrichment in the two clusters. **(F)** Immunophenoscore for predicting the responsiveness to CTLA-4 checkpoint inhibitors and PD-1 checkpoint inhibitors. CTLA-4: Cytotoxic T-lymphocyte associated protein 4; PD-1: Programmed cell death 1. (*P <0.05, **P < 0.01, ***P < 0.001, ns: Not statistically significant).

The heatmap shown in **Figure 3A** distinctly divided patients into two clusters based on m7G-related gene expression data. These gene comparisons between clusters showed that most of the genes were highly expressed in cluster B compared to cluster A, such as CYFIP1, DCP2, EIF3D, EIF4E, and WDR4 (**Figure 3B**). Kaplan–Meier plot showed that cluster B was associated with worse survival than cluster A (**Figure 3C**). On the other hand, a single-sample GSEA for immune cell infiltration analysis was performed, and the results were available in **Supplementary Table S1**. Then, the results of the comparison of immune infiltration between clusters were visualized in the boxplot, which showed that infiltration levels of most types of immune cells were downregulated in cluster B (**Figure 3D**). GSVA results on KEGG pathways were visualized in **Figure 3E** by a heatmap, which showed pathways enriched in cluster B including cell cycle, homologous recombination, spliceosome, nucleotide excision repair, and mismatch repair, while cluster A was enriched in graft-versus-host disease, transduction, intestinal immune network for IgA production, asthma, allograft rejection, autoimmune thyroid disease pathways, and so on. Moreover, the IPS for prediction responsiveness to CTLA-4 and PD-1 checkpoint inhibitors was also compared between m7G clusters, which showed that the responses to all therapies were always lower in cluster A group (**Figure 3F**) whether using CTLA-4 checkpoint inhibitors or PD-1 checkpoint inhibitors or both.

### An m7G-related prediction model was constructed by Cox regression

**Figure 4:**
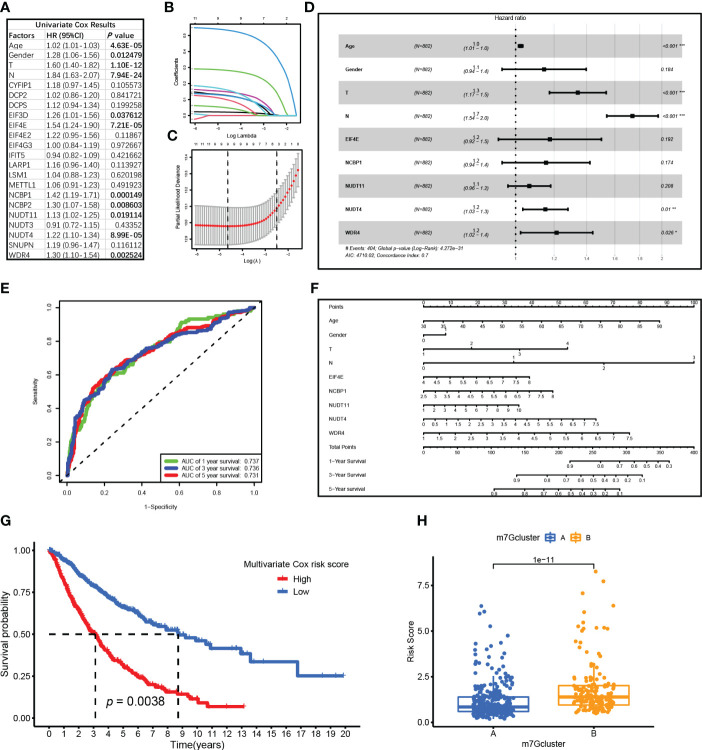
Cox regression analysis for N7-methylguanosine (m7G)-related genes and clinical characteristics. **(A)** Univariate Cox regression analysis results of m7G-related genes. **(B)** Coefficient plot for each factor coefficient. **(C)** The partial likelihood deviance plot showed partial likelihood deviance of each model. **(D)** Forest plot showed multivariate Cox regression analysis results of the nine factors. **(E)** ROC curve showed the survival prediction model performance. **(F)** The nomogram for survival prediction model. **(G)** Survival analysis between high-risk and low-risk groups. **(H)** Boxplot showed Cox risk scores between the two m7G clusters. ROC: Receiver operating characteristic curve; AUC: Area under curve. (*P <0.05, **P < 0.01, ***P < 0.001)

A total of 882 patient samples with clinical characteristics were included in the analysis. First, the univariate Cox regression analysis results were shown in **Figure 4A**, and 11 factors were potential risk factors to patients, namely, age (*P* = 4.63E-05, HR = 1.02), gender (*P* = 0.012, HR = 1.28), T stage (*P* = 1.10E-12, HR = 1.60), N stage (*P* = 7.94E-24, HR = 1.84), EIF3D (*P* = 0.038, HR = 1.26), EIF4E (*P* = 7.21E-05, HR = 1.54), NCBP1 (*P* < 0.001, HR = 1.42), NCBP2 (*P* = 0.009, HR = 1.30), NUDT11 (*P* = 0.019, HR = 1.13), NUDT4 (*P* = 8.99E-05, HR = 1.22), and WDR4 (*P* = 0.003, HR = 1.30). Then, these risk factors were included in the LASSO Cox analysis for optimizing the most fitted model. The coefficient plot showed each factor coefficient using different color curves (**Figure 4B**), in which genes EIF3D (the fifth curve in the plot) and NCBP2 (the eighth curve in the plot) represented lower coefficients, and the cross-validation curve showed that the models that contained the remaining nine factors were efficient enough to minimize partial likelihood deviance (**Figure 4C**); thereupon, the genes EIF3D and NCBP2 were excluded in the following analysis. At last, the remaining nine factors were included in the multivariate Cox regression analysis, and the results were visualized by a forest plot in **Figure 4D**. It turns out that factors including age, T stage, N stage, NUDT4, and WDR4 are independent risk factors in LUAD patients. The ROC curve showed that the model possesses a good performance in predicting patients’ survival, with an area under the curve (AUC) of 0.737, 0.736, and 0.731 for 1-year, 3-year, and 5-year survival, respectively (**Figure 4E**). The nomogram for the survival prediction model was plotted that can be used to manually obtain predicted risk values according to the risk factors from the regression model (**Figure 4F**). The risk score was calculated by multivariate Cox analysis, available in **Supplementary Table S2**. The high-risk group logically correlated with a poor prognosis (**Figure 4G**). On the other hand, we also compared risk scores between the two clusters, in which cluster B possessed higher risk scores compared to those in cluster A (**Figure 4H**).

### A negative correlation tendency between m7G and immune infiltration was also observed in single-cell analysis

**Figure 5:**
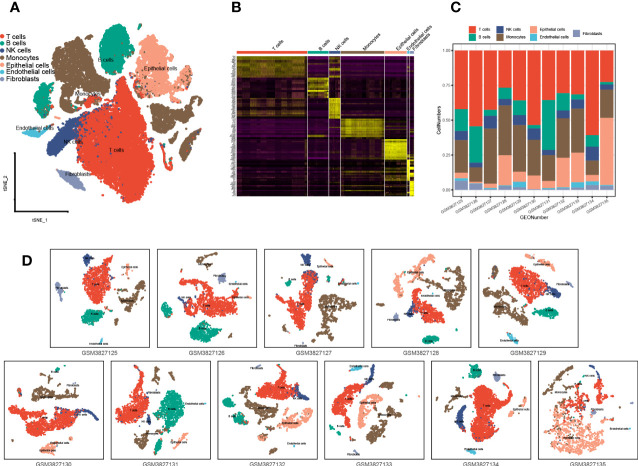
Single-cell analysis and cell type identification for 11 lung adenocarcinoma patient samples. **(A)** Seven main cell types of all 11 samples were integrated in one Seurat project and visualized in t-SNE plot. **(B)** The top 10 marker genes of different cell types were visualized in the heatmap. **(C)** Cell type proportions in different patient samples. **(D)** Seven main types of cells in different patient samples were visualized in the t-SNE plot. t-SNE: t-Distributed Stochastic Neighbor Embedding.

**Figure 6:**
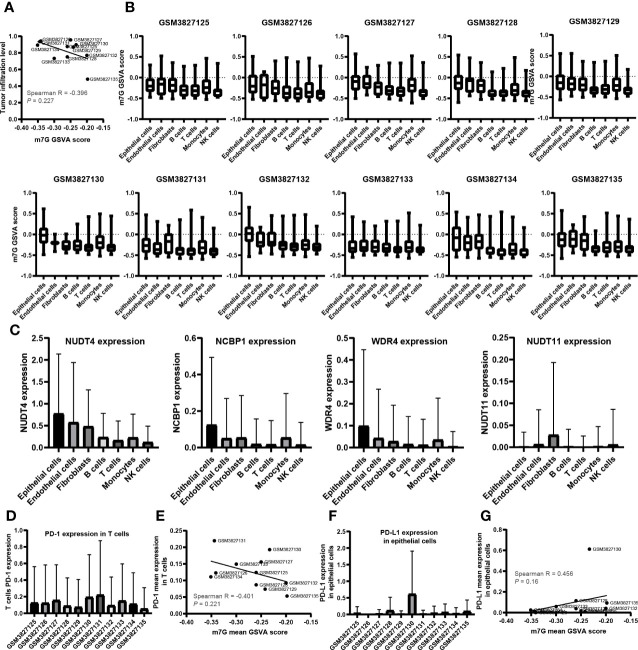
The relationship between N7-methylguanosine (m7G) GSVA scores and tumor immune infiltration cells. **(A)** The relationship between m7G mean GSVA scores and tumor immune infiltration levels. **(B)** m7G mean GSVA scores in different cell types and different samples. **(C)** The expression level of Cox independent risk factors in different cell types. **(D)** PD-1 expression of T cells in different samples. **(E)** The relationship between m7G mean GSVA scores and T cell mean PD-1 expression. **(F)** PD-L1 expression of epithelial cells in different samples. **(G)** The relationship between m7G mean GSVA scores and epithelial cell PD-L1 mean expression. GSVA: Gene set variation analysis; PD-1: Programmed cell death 1; PD-L1: Programmed cell death 1 ligand 1.

A total of 43,025 cells, with seven main cell types identified in the analysis, namely, T cells (n = 17,420), monocytes (n = 10,690), B cells (n = 5,145), NK cells (n = 2,764), endothelial cells (n = 635), fibroblasts (n = 961), and epithelial cells (n = 5,410), contained tumor cells (**Figure 5A**), and the top 10 marker genes in the different cell types were shown in the heatmap (**Figure 5B**). There are various tumor infiltration levels in the 11 samples (**Figure 5C**), namely, GS3827125 (stage I), GSM3827126 (stage I), GSM3827127 (stage II), GSM3827128 (stage I), GSM3827129 (stage I), GSM3827130 (stage I), GSM3827131 (stage I), GSM3827132 (stage III), GSM3827133 (stage I), GSM3827134 (stage III), and GSM3827135(stage I), and all cell types in each sample were visualized using t-Distributed Stochastic Neighbor Embedding t-SNE scatterplot (**Figure 5D**). GSVA for m7G-related genes was available in **Supplementary Table S3**, and in **Figure 6A**, mean GSVA scores in different samples had a negative correlation tendency with immune infiltration levels but were not statistically significant (R = -0.396, *P* = 0.227). Otherwise, high GSVA scores were almost all gathered in epithelial cell groups in all samples (**Figure 6B**). Independent risk factors that we analyzed in Cox regression were also analyzed in single-cell data, and NUDT4, NCBP1, and WDR4 were mostly expressed in tumor cells (**Figure 6C**); other m7G-related genes expression data were available in **Supplementary Table S4**. On the other hand, to verify TCIA IPS results, we analyzed PD-1 expression in T cells (**Figure 6D**), which also had a negative correlation tendency with m7G GSVA scores but was not statistically significant (R = -0.401, *P* = 0.221) (**Figure 6E**), and PD-L1 expression in epithelial cells (**Figure 6F**) had a positive correlation tendency with m7G GSVA scores and still not statistically significant (R = 0.456, *P* = 0.16) (**Figure 6G**).

### NUDT4 expression analysis in bulk RNA level, protein level, and single-cell RNA level

**Figure 7:**
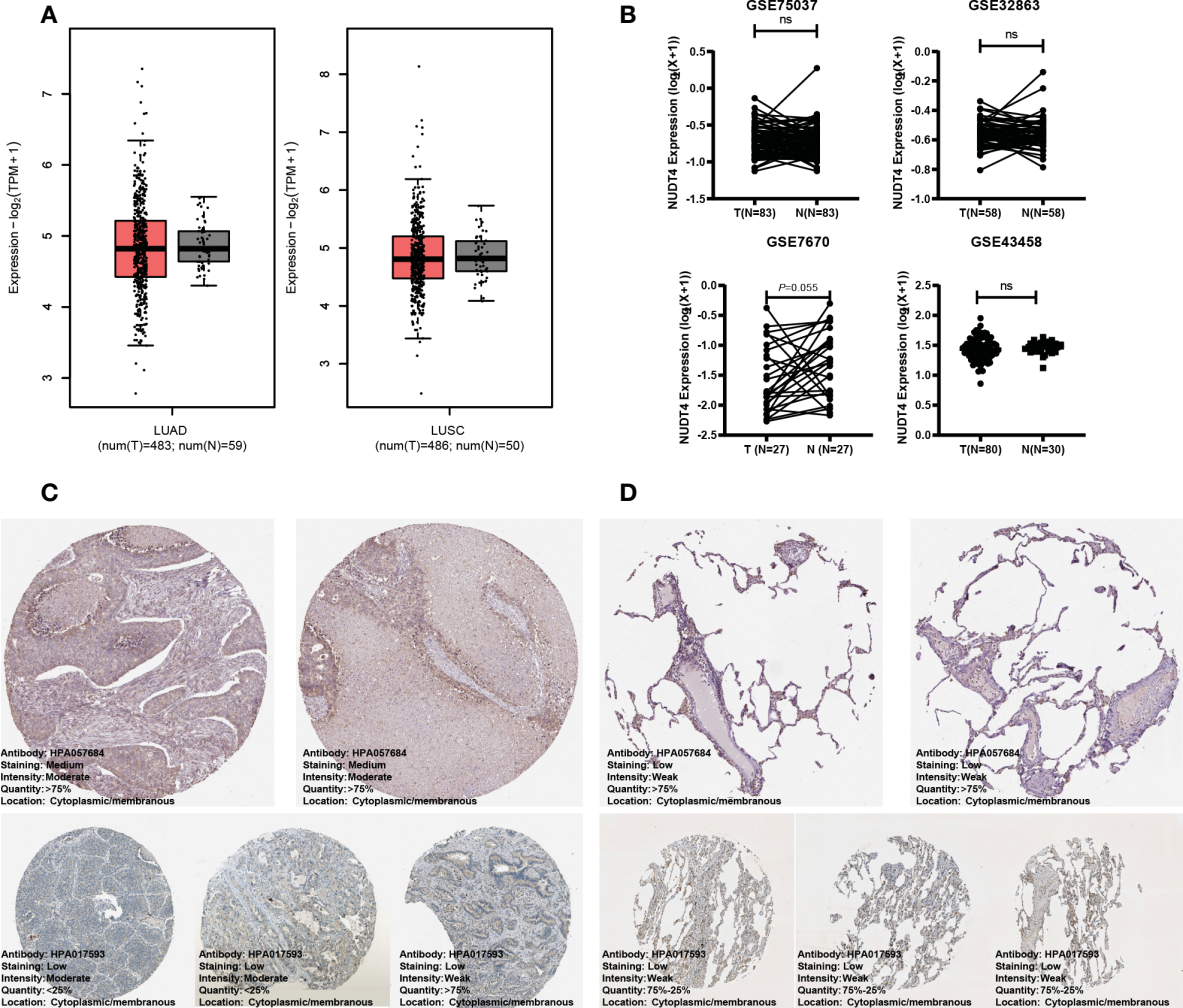
NUDT4 expression in bulk RNA level and protein level. **(A)** NUDT4 expression in lung adenocarcinoma and squamous carcinoma in TCGA datasets. **(B)** NUDT4 expression in lung adenocarcinoma in GEO datasets. **(C)** The protein level of NUDT4 in lung adenocarcinoma chips using immunochemistry. **(D)** The protein level of NUDT4 in normal lung tissue chips using immunochemistry. LUAD: Lung adenocarcinoma; LUSC: Lung squamous cell carcinoma; ns: Not statistically significant.

**Figure 8:**
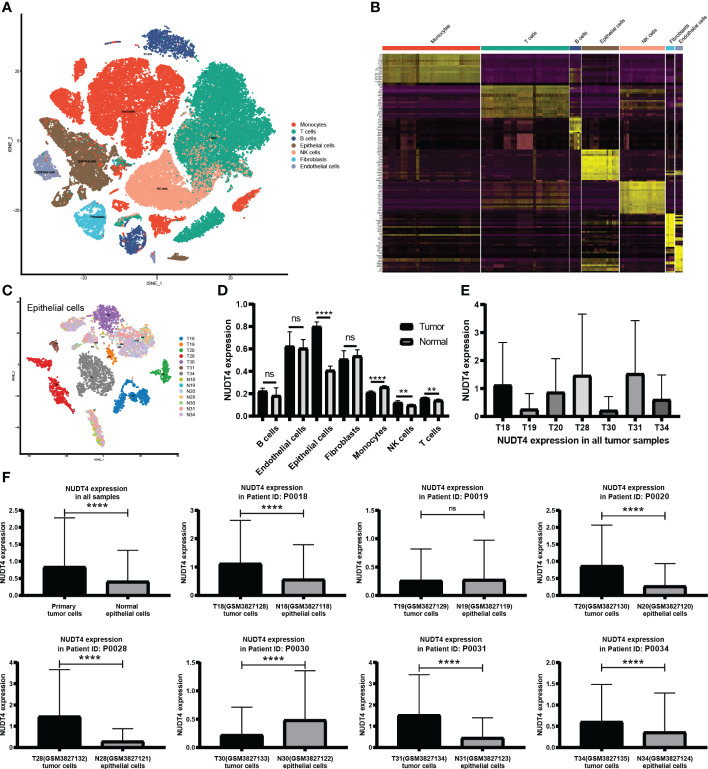
NUDT4 expression in single-cell RNA level. **(A)** Seven main cell types of tumor and normal samples were integrated in one Seurat project and visualized in t-SNE plot. **(B)** The top 10 marker genes of seven cell types. **(C)** Only epithelial cells were selected for NUDT4 analysis. **(D)** NUDT4 expression in different cell types. **(E)** NUDT4 expression in different samples. **(F)** NUDT4 expression in paired tumor and normal derived epithelial cells. (**P< 0.01, ****P < 0.0001, ns: Not statistically significant).

As mentioned above, NUDT4 was an independent risk factor in lung cancer patients (**Figure 4D**), and there is no research exploring its function in lung cancer. To further confirm its potential role in lung cancer, we analyzed its expression level using TCGA bulk RNA data and GEO datasets. However, there is no difference between tumor and normal tissues either in LUAD or in squamous cancer subtypes (**Figures 7A, B**). But when we compared immunochemistry results, the NUDT4 was found to be expressed more in tumor chips than normal tissue chips (**Figures 7C, D**). Therefore, to more specifically understand its expression between cancer and normal cells, we integrated tumor and normal single-cell RNA data (**Figures 8A, B**) and intend to select epithelial cells for analysis (**Figure 8C**). Among the seven cell types with NUDT4 expression, there is a significant difference in epithelial cells between tumor and normal samples (**Figure 8D**) but varies in different patients (**Figure 8E**). In paired patients’ samples, NUDT4 was highly expressed in tumor epithelial cells of six patients (**Figure 8F**).

### NUDT4 was involved in tumor cell proliferation but not in migration

**Figure 9:**
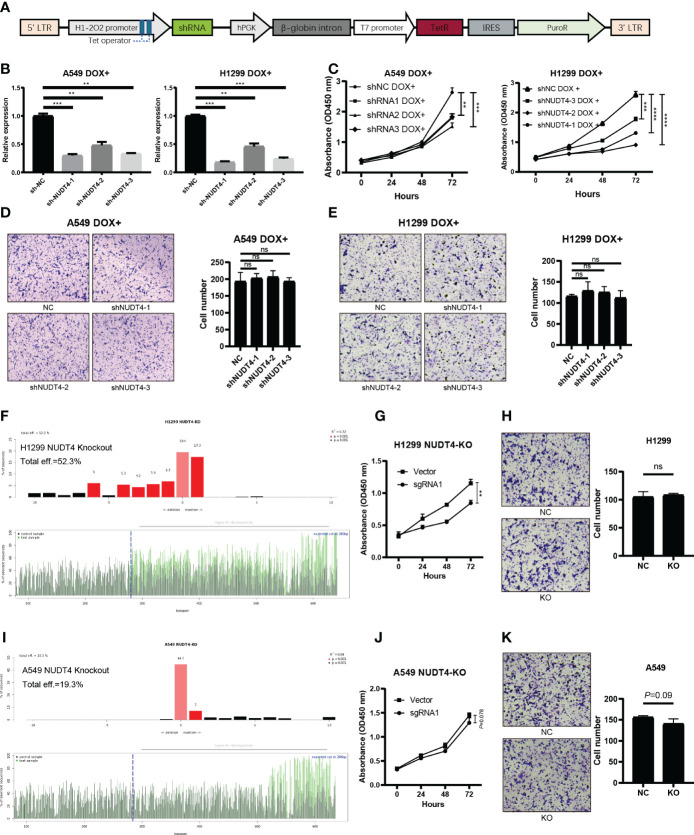
NUDT4 function validation in lung adenocarcinoma cell lines. **(A)** Sequence map of tet-on system shRNA plasmids. **(B)** NUDT4 knocking down efficiency in cell lines. **(C)** CCK8 assay for detecting cell proliferation (n = 3 duplicates). **(D)** Transwell assay for detecting A549 cell migration (n = 3 duplicates). **(E)** Transwell assay for H1299 cell migration capability detection (n = 3 duplicates). **(F)** Knockout efficiency of the NUDT4 in H1299 cell line. **(G)** CCK8 assay for detecting H1299 cell proliferation (n = 3 duplicates). **(H)** Transwell assay for detecting H1299 cell migration (n = 3 duplicates). **(I)** Knockout efficiency of the NUDT4 in A549 cell line. **(J)** CCK8 assay for detecting A549 cell proliferation (n = 3 duplicates). **(K)** Transwell assay for detecting A549 cell migration (n = 3 duplicates). (**P < 0.01, ***P < 0.001, ****P < 0.0001, ns: Not statistically significant).

Then, to explore its function in tumor cells, we designed shRNAs, sgRNAs, and overexpression plasmids to knock down, knock out, or overexpress NUDT4 expression. However, during the first time, NUDT4 expression was knocked down, but cells could not be amplified and used in the following experiments. So, we cloned shRNA sequence to Tet-on system plasmids (**Figure 9A**), and when stable cell lines were constructed, doxycycline was used to induce cell shRNA expression, then the knockdown efficiency was verified by RT-qPCR (**Figure 9B**). NUDT4 knockdown inhibiting cell proliferation was observed in both A549 and H1299 cells (**Figure 9C**). But there seems to be no difference in migration capability between groups (**Figures 9D, E**).

**Figure 10:**
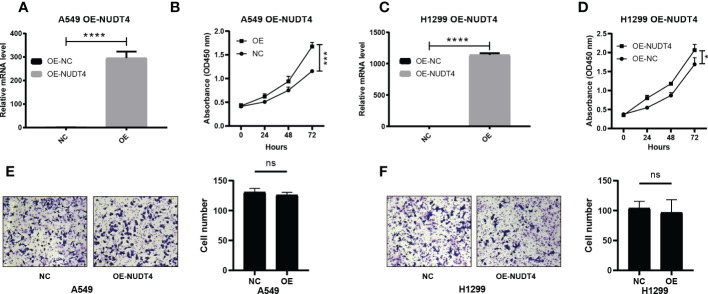
Overexpression of NUDT4 in lung adenocarcinoma cell lines. **(A)** Overexpression level in A549 cell line. **(B)** CCK8 assay for detecting cell proliferation (n = 3 duplicates). **(C)** NUDT4 overexpression level in H1299 cell line. **(D)** CCK8 assay for observing H1299 cell proliferation (n = 3 duplicates). **(E)** Transwell assay for A549 cell capability of the migration (n = 3 duplicates). **(F)** Transwell assay for detecting H1299 cell migration (n = 3 duplicates). (*P < 0.05, ***P < 0.001, ****P < 0.0001, ns: Not statistically significant).

To further verify the results on NUDT4 DNA level, we also designed sgRNAs to try to knock out NUDT4 exons. The knockout efficiency was analyzed by TIDE (https://tide.nki.nl/), which showed that only sgRNA1 worked in H1299 cell line (knockout efficiency = 52.3%, **Figure 9F**) but did not work well in A549 cell line (knockout efficiency = 19.3%, **Figure 9I**). Cell proliferation and migration capabilities were also determined, which showed similar results compared with shRNAs (**Figures 9G–K**). On the other hand, we also cloned NUDT4 mRNA into A549 and H1299 cell lines to overexpress NUDT4 and performed RT-qPCR to verify the overexpression efficiency (**Figures 10A, C**). Then, the CCK8 assay (**Figures 10B, D**) and Transwell assay were conducted to observe the cell functions, which showed that overexpression of the NUDT4 promoted cell proliferation capabilities in both cell lines, but the migration capabilities of the tumor cells were still untouched (**Figures 10E, F**).

## Discussion

The number of lung cancer patients diagnosed with the pathological subtype adenocarcinoma was accumulating in the past years, especially in China and East Asia (44). As low-dose CT screening was widely included in human health examination, patients in early stages were screened out and therefore reduced the mortality rate (45), but patients with advanced stages of LUAD still struggled. Although tyrosine kinase inhibitors made great progress in alleviating tumor progression and clinical symptoms, tumor recurrence nearly inevitably occurred within 1 or 2 years (46). In recent years, immunotherapy-based drugs such as immune checkpoint inhibitors had been reported to make a huge progression in improving survival in patients with advanced lung cancer, which worked by boosting immune cell cytotoxicity to kill the tumor cells (47). However, the response rate to immunotherapy is approximately 20%, and there were no efficient biomarkers able to distinguish who among the patients responded to immunotherapy or not (48, 49). It was reported that RNA modification can regulate tumor immunity, which has the potential to provide us with new biomarkers to screen who among the patients were sensitive to immunotherapy and even identify new immunotherapy targets (50).

In this study, we focused on exploring the potential role of m7G modification in LUAD. First, genetic variations were evaluated; genes AGO2, NSUN2, and METTL1 were observed to be the most amplified in the results. It was reported that AGO2, as a component of the RNA-induced silencing complex, was capable of inhibiting protein translation by binding to the m7G cap of EIF4E (51), which was essential in the translational process of tumor progression (52). According to these pathways, AGO2 may act as a tumor suppressor in lung cancer patients, which contradicted its high amplification rate in our results. But another study demonstrated that AGO2 could physically interact with RAS (HRAS, NRAS, and KRAS) and activated KRAS (GTPase) signaling to promote lung cancer progression (53), which was consistent with our results that AGO2 may act as a tumor promoter in LUAD patients and may be a potential target for treatment. In addition, RNA methyltransferase NSUN2, capable of adding m5C to mRNA (54), was reported to modify the effect of T-cell activation on patient survival in head and neck squamous carcinoma (55). But the mechanical explorations for NSUN2 in lung cancer were unclear and need further study. METTL1 was a methyltransferase catalyzing m7G modification of tRNAs, and METTL1-mediated tRNA modification drives oncogenic transformation (56) and promotes lung cancer progression (16). On the other hand, mutation status results showed that the top mutated genes such as EIF4G3, which was reported to silence EIF4G3, could induce cell apoptosis and suppress tumor growth in lung cancer cell lines (57). But there is no statistical difference in patients’ survival between mutant and wild-type groups, which may impute to the insufficient number of mutant samples.

In the second part of our study, m7G-related gene mRNA comparisons between tumor and normal samples were performed, in which METTL1, NSUN2, WDR4, EIF3D, NCBP1, EIF4E, LARP1, and LSM1 were significantly upregulated in tumor samples. NCBP1 is essential for capped RNA processing and intracellular localization, which was reported to promote lung cancer progression and epithelial–mesenchymal transition *via* NCBP1-NCBP3-CUL4B oncoprotein axis (58). In our results, NCBP1 was associated with poor survival outcomes in LUAD patients. Similarly, highly expressed LSM1 was also reported to promote tumor cell growth in epithelial tissues, contributing to the transformed state (59). LARP1 is an RNA-binding protein that interacts with poly-A-binding protein and was reported to function as an oncogene to promote lung cancer cell growth, migration, and invasion (60). However, there were a few downregulated genes in tumor samples, such as CYFIP1, IFIT5, DCP2, and EIF4E3. In prostate and bladder cancer, it was reported that IFIT5 was able to promote epithelial–mesenchymal transition and progression, but there is no study reported in lung cancer, so the role of IFIT5 in lung cancer still needs to be explored (61, 62).

Unsupervised consensus clustering divided samples into two clusters, and results showed a higher expression of m7G genes and poor survival in cluster B, which were consistent with the former single gene survival analysis. In addition, immune infiltration results showed a low-level infiltration in cluster B. RNA modification process impacts the tumor immune microenvironment and regulates tumor development (63). It was reported that ALKBH5 and m6A demethylase deletion increased tumor cell sensitivity to immunotherapy and that m6A demethylases in tumor cells contribute to the efficacy of immunotherapy (64). In pancreatic adenocarcinoma, m6A-related genes arm-level gain and deletion decreased the infiltration of CD8+ T cells (65), which was consistent with our results that m7G-related genes were associated with decreased infiltration of multiple types of immune cells. Pathways enriched in cluster A included an intestinal immune network for IgA production, asthma, autoimmune thyroid disease, and allograft rejection. All of these pathways were closely related to immune responses, which was consistent with a high immune infiltration level and might indicate that the tumor environment in cluster A has high immunological competence. Multivariate Cox regression analysis showed that genes NUDT4 (*P* = 0.01, HR = 1.2) and WDR4 (*P* = 0.026, HR = 1.2) were independent risk factors. In our results, NUDT4 was related to poor survival, but little is known in cancer development. WDR4 combined with METTL1 as m7G methyltransferase complex components, and it had been reported that depletion of METTL1 and WDR4 resulted in decreased lung cancer cell progression (16). TCIA is based on the expression of gene sets including MHC (Major histocompatibility complex) molecules (such as B2M, HLA-A, HLA-B), immunomodulators (such as PD-1, CTLA-4, LAG3 (Lymphocyte activating 3), effector cells (such as activated CD8+ T cells and CD4+ T cells), and suppressor cells (myeloid-derived suppressor cells and Tregs (Regulatory T cells). And was a superior predictor of response to CTLA-4 and PD-1 antibodies (66). In our results, the IPS in the cluster B group was significantly lower than that in the cluster A group, which could be speculated that high m7G modification status may correspond to poor responses to immunotherapy. Combined with single-cell analysis results, we also observed the same tendency of PD-1, although the statistics were not significant, which may result from the shortage of sample sizes.

There is no research studying the potential function of NUDT4 in lung cancer until now, and we observed its capability in promoting cancer cell proliferation but not cell migration according to our Transwell results. How NUDT4 affects tumor proliferation and what pathway is involved in this phenomenon need further exploration.

## Conclusions

In summary, using a comprehensive analysis of three different types of datasets, our study analyzed m7G-related genes in LUAD and correlated them with clinical characteristics and immune infiltration. We also constructed a prognostic model based on m7G genes and the risk score for predicting the survival outcome of patients. On the other hand, we found that NUDT4 might be a novel target inhibiting tumor cell proliferation.

## Data availability statement

The original contributions presented in the study are included in the article/**Supplementary Material**. Further inquiries can be directed to the corresponding author.

## Author contributions

MW conceived, designed, and supervised the study. YL, CL, and BJ drafted the manuscript. ZL, ZJ, and CW collected the data. YL, CL, and BJ performed all data analysis. All authors contributed to the article and approved the submitted version.
